# Evaluating the Impact of PSA as a Selection Criteria for Nerve Sparing Radical Prostatectomy in a Screened Cohort

**DOI:** 10.1155/2014/395078

**Published:** 2014-04-16

**Authors:** Shyam K. Tanguturi, Ming-Hui Chen, Marian Loffredo, Jerome P. Richie, Anthony V. D'Amico

**Affiliations:** ^1^Harvard Radiation Oncology Program, Harvard Medical School, Boston, MA 02115, USA; ^2^Brigham and Women's Hospital, 75 Francis Street, ASB1 L2, Boston, MA 02115, USA; ^3^Department of Statistics, University of Connecticut, Storrs, CT 06269, USA; ^4^Lank Center for Genitourinary Oncology, Dana Farber Cancer Institute, Boston, MA 02115, USA; ^5^Department of Radiation Oncology, Dana Farber Cancer Institute and Brigham and Women's Hospital, Boston, MA 02115, USA

## Abstract

*Purpose*. We investigated whether NS-RP increased risk of PSA failure and whether PSA should be included as a selection criterion for NS. *Methods*. We evaluated 357 consecutive men with screen-detected PC who underwent open RP without adjuvant radiotherapy between 9/11/2001 and 12/30/2008. Criteria for NS included Gleason score ≤3 + 4, percentage of positive biopsies (PPB) ≤50%, percentage of core involvement ≤50%, nonapical location, no perineural invasion, and no palpable disease on pre- or intraoperative exam but did not include a PSA threshold. Cox multivariable regression assessed whether increasing PSA or unilateral- or bilateral-NS versus non-NS-RP was associated with PSA failure adjusting for prognostic factors. *Results*. After a median follow-up of 3.96 years, 34 men sustained PSA failure (9.5%). Increasing PSA was significantly associated with increased risk of PSA failure in the interaction model (adjusted hazard ratio (AHR): 1.09 [95% CI: 1.03–1.16]; *P* = 0.005), whereas unilateral (AHR: 1.24 [95% CI: 0.36–4.34]; *P* = 0.73) or bilateral NS (AHR: 0.41 [95% CI: 0.06–2.59]; *P* = 0.34) versus non-NS RP was not. *Conclusion*. NS-RP in a screened cohort did not increase risk of PSA failure using NS criteria not including PSA.

## 1. Introduction

Nerve-sparing (NS) radical prostatectomy (RP) was introduced in 1984 by Dr. Patrick Walsh as a means of preserving potency and maintaining patient reported health related quality of life following NS RP [1, 2]. This procedure involves preservation of the neurovascular bundles (NVB), which travel dorsolateral to the prostate and are believed to supply neurophysiologic control of erectile tissue [3]. NS RP has been found to correlate with early return of urinary continence when applied in a risk-stratified approach [4], which is another potential benefit of this approach.

Since that time, the procedure has been widely used, with rates of patient reported postoperative potency ranging from 21 to 89.7% after RP [5–11]. Despite the wide adoption of NS RP, prospective randomized assessment of prostate cancer control outcomes with or without NS is lacking. Some have raised the concern that this procedure may place select patients with large tumor burden at increased risk of post-RP prostate-specific antigen (PSA) failure by increasing the risk of positive surgical margins due to the close proximity of the NVBs to the posterolateral aspect of the prostatic fascia [12–14]. However, this concern has been debated [12, 15, 16].

Different investigators have attempted to define tumor factors believed to optimize selection for NS to thereby reduce the risk of a positive margin [12, 17, 18]. Earlier series from the Washington University Medical Center describe ideal candidates for NS RP as young patients with clinical stage not exceeding T2 and well-differentiated tumors [18]. An algorithm from New York University (NYU) reserves NS RP for patients with Gleason 6 or less tumors with percentage of positive biopsies (PPB) less than 50% or no perineural invasion (PNI), Gleason 7 tumors with PPB less than 30% and no PNI, and Gleason 8 to 10 tumors with PPB less than 10% and no PNI [17]. At the University of Miami, Florida, similar criteria include preoperative potency, desire to maintain potency, biopsies with tumor on one side only, T2b disease or less, intraoperative absence of palpable induration or periprostatic fibrosis, Gleason score of 7 or less, PSA of 10 ng/mL or less, and nonapical location of tumor [12, 16].

At the Brigham and Women's Hospital, criteria for NS by side included Gleason score 3 + 4 or less, PPB and percentage of core involvement 50% or less, nonapical location, and no perineural invasion or palpable disease on pre- or intraoperative exam but did not include a maximum PSA value. Therefore using these criteria as the basis for NS or not, the current study was designed to investigate whether an increasing PSA level and whether unilateral (UNS) or bilateral (BNS) versus non-NS RP were associated with an increased risk of PSA failure adjusting for known predictors for PSA recurrence in men who did not undergo adjuvant radiation therapy.

## 2. Patients and Methods

### 2.1. Patient Population and Treatment

We identified 357 consecutively treated men with screen-detected biopsy proven prostate cancer (PC) who underwent an open radical prostatectomy by a single urologic oncologist between 9/11/2001 and 12/30/2008 at a single academic center. Determination of the use of bilateral and unilateral NS was judged on each side separately and was based on the presence of Gleason score 3 + 4 or less, PPB and percentage of core involvement 50% or less, nonapical location, and no perineural invasion or palpable disease on pre- or intraoperative exam but did not include a maximum PSA value.

### 2.2. Pathologic Processing of the RP Specimen

RP pathologic specimens were step-sectioned and reviewed by an expert genitourinary pathologist as previously described [19]. The apical and basal margins were amputated to a thickness of 5 mm and sectioned at 3 mm intervals parasagittally or perpendicular to the initial transverse incision. The base of the seminal vesicles was amputated and the basal cross-section submitted. The prostate was sectioned as thinly as possible perpendicularly to the long axis (apical to basal) of the gland, typically at 3 mm intervals, with most specimens requiring four to seven cross-sections. For each cross-section, a single section each of the right and left posterior region was submitted, with most cases entirely submitting the posterior zone. Finally, at least one section of the mid-anterior prostate was also submitted, although frequently more than one section was submitted for histology analysis.

Evidence of extraprostatic disease including seminal vesicle invasion (SVI), extracapsular extension (ECE), nodal involvement (pN1), and/or positive surgical margins was recorded, and tumor staging was defined using the 2010 American Joint Committee on Cancer (AJCC) categorization. All biopsy and prostatectomy Gleason scores were centrally reviewed and determined using a primary and secondary Gleason grade +/− a tertiary pattern in a manner similar to that outlined in the 2005 International Society of Urological Pathology (ISUP) consensus recommendations [20]. This study was approved by the institutional review board at the Brigham and Women's Hospital.

### 2.3. Follow-Up and Determination of PSA Failure

Adjuvant radiation therapy was not used. Patient follow-up consisted of a serum PSA measurement and digital rectal examination every 3 months for the first 2 years, every 6 months for the next 3 years, and annually thereafter. PSA failure was defined in accordance with American Urological Association (AUA) guidelines as a postoperative serum PSA level greater than 0.2 ng/mL, confirmed on a second measurement at least one month later [21].

### 2.4. Statistical Methods

#### 2.4.1. Comparison of the Distribution of Clinical and Pathologic Factors in Men Selected for Bilateral or Unilateral versus Non-Nerve Sparing Radical Prostatectomy

The distribution of PSA, PPB, age, clinical tumor category, biopsy and prostatectomy Gleason score, and prostatectomy tumor category and margin status was compared across men who had bilateral, unilateral, or non-NS RP. The distributions of these characteristics were compared using a Mantel-Haenszel Chi Squared metric for categorical factors and [22] a nonparametric Wilcoxon statistic for continuous covariates [23] (Table 1). In the case of small sample size, a Fisher's exact test was employed [22].

#### 2.4.2. Time to PSA Failure Analyses

A Cox Proportional Hazards multivariable regression model was used to evaluate whether increasing PSA or use of UNS or BNS versus non-NS RP was associated with the risk of PSA failure after adjusting for known predictors of PSA failure, including an interaction term between PPB and PSA in the absence of adjuvant radiation therapy use [24]. Because PPB was used whereas PSA level was not used as a selection criterion for NS RP and because both PSA and PPB are associated with tumor volume, which may in turn increase the risk of positive surgical margins and subsequent PSA failure following NS RP, we included an interaction term in the model between PSA and PPB. Other variables included in the multivariable model included age, PSA and PPB (both continuous) at diagnosis, Gleason Score (8–10 versus 7 versus 6 or less as baseline), tumor category (cT2-3 versus T1c as baseline), and type of NS RP (UNS or BNS versus non-NS as baseline). Unadjusted and adjusted hazard ratios with 95% confidence intervals and associated *P* values were calculated for each covariate. A 2-sided *P* value < 0.05 was considered statistically significant. SAS version 9.3 was used for all statistical analyses (SAS Institute, Inc. Cary, NC).

#### 2.4.3. Estimates of PSA Failure

The Kaplan-Meier method was used to estimate PSA failure-free survival for men following RP stratified by whether bilateral, unilateral, or non- NS was employed [7]. Comparison of these Kaplan-Meier estimates was performed using a log rank test [25]. A Bonferroni correction was applied to adjust for multiple comparisons (*n* = 3), such that a significant *P* value was <0.05/3 or 0.0167 [26]. Point estimates of PSA failure-free survival with associated 95% confidence intervals were calculated and reported.

## 3. Results

### 3.1. Comparison of the Distribution of Clinical and Pathologic Factors in Men Selected for Bilateral or Unilateral versus Non-Nerve Sparing Radical Prostatectomy

As shown in Table 1, men undergoing BNS compared to UNS and non-NS were significantly younger and had more favorable disease characteristics including median PPB, clinical tumor category, biopsy Gleason score, as well as prostatectomy findings of tumor category, Gleason score, and margin status (all *P* values for trend < 0.0001). However, median PSA was not significantly different between men undergoing BNS, UNS, or non-NS, respectively, 4.6 ng/mL, 5.0 ng/mL, and 4.6 ng/mL (*P* value for trend = 0.137). Overall, 7% of men in this study had a PSA of 10 ng/mL or more.

As indicated by the distribution of BNS, UNS, non-NS across PPB and PSA categories, Table 1 also illustrates that PPB was used as a selection criteria for NS whereas PSA level was not. Specifically, high PPB (>median of 25%) led to lower rates of BNS between 25 and 33%, whereas high PSA (>median of 4.8 ng/mL) did not correlate to increased or decreased use of BNS ranging from a high of 68% when the PPB was ≤median to a low of 25% when the PPB was greater than the median. Among men with low PPB (<median), rates of BNS remained high at 68% and 74% irrespective of whether the PSA level was greater than median or less than median, respectively.

### 3.2. Time to PSA Failure Analysis

After a median follow-up of 3.96 years (interquartile range 1.92–5.00), 34 out of 357 men sustained PSA failure (9.5%). While increasing PSA was significantly associated with an increased risk of PSA failure in the interaction model (adjusted hazard ratio (AHR): 1.09 [95% Confidence Interval (CI): 1.03 to 1.16]; *P* = 0.005) the use of UNS (AHR: 1.24 [95% CI: 0.36 to 4.34]; *P* = 0.73) or BNS (AHR: 0.41 [95% CI: 0.06–2.59]; *P* = 0.34) versus non-NS RP was not.

Other factors significantly associated with an increased risk of PSA failure included increasing PPB (AHR: 1.03 [95% CI: 1.01 to 1.05]; *P* = 0.009), biopsy Gleason score 7 (AHR: 9.70 [95% CI: 2.17 to 43.37]; *P* = 0.003), and biopsy Gleason score 8–10 (AHR: 25.01 [95% CI: 5.08 to 123.14]; *P* < 0.001) compared to 6 or less.

### 3.3. Estimates of PSA Failure

**Figure 1:**
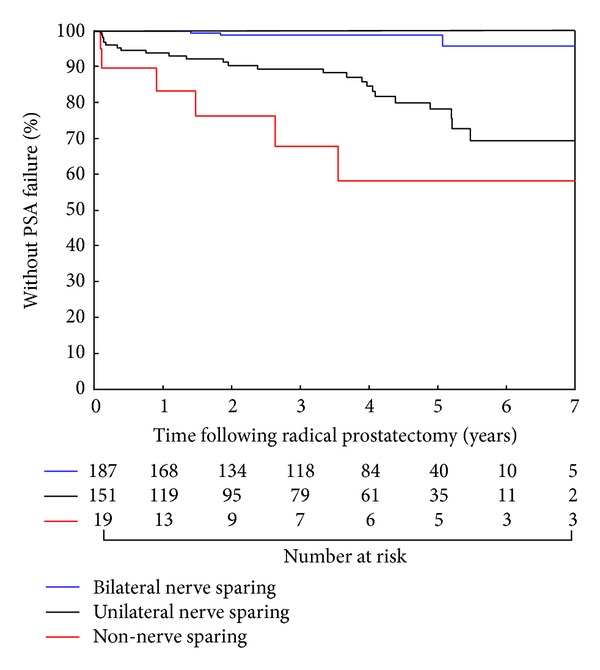
PSA failure-free survival following radical prostatectomy, stratified by whether one, both, or neither nerve was spared.

As shown in Figure 1, the univariable estimates of PSA failure-free survival decreased significantly in men undergoing non-NS as compared to UNS as compared to BNS (overall log-rank test *P* value < 0.0001). Specifically, 4-year point estimates of PSA failure-free survival for men undergoing BNS, UNS, and non-NS were 98.7% (95% CI: 94.9 to 99.7%), 84.4% (95% CI: 75.9 to 90.0%), and 58.0% (95% CI: 27.7 to 79.4%), respectively.

## 4. Discussion

In this study, we found that men undergoing UNS or BNS versus non-NS had more favorable prognostic factor distributions (Table 1) with respect to PPB, Gleason score, and T category but not PSA level, which is consistent with the selection criteria used for NS. This is in turn was reflected in more favorable PSA outcomes among men undergoing UNS or BNS versus non-NS RP, as illustrated in Figure 1. However, after adjusting for prognostic factors in the multivariable model, the use of UNS or BNS as compared to non-NS no longer predicted for a lower risk of PSA failure (Table 2), despite the lack of use of adjuvant radiation therapy. Together, these data support that the selection criteria used for NS in this study are effective and do not place men at increased risk of PSA failure.

Nevertheless, some investigators have published that a PSA < 10 ng/mL and no more than one high-grade core biopsy predicts for ipsilateral organ confined disease at a rate of 88.5% [27]. Therefore some have advocated for use of PSA < 15 ng/mL in addition to diffusion weighted MRI, PPB, and T category to select patients for NS RP [28]. Results in the current study cannot be used to design the optimal cut-point (i.e., <10 or <15 ng/mL), given that 93% of men in this cohort had a PSA < 10 ng/mL. While the results of this study support the hypothesis that the use of UNS or BNS did not impact the increased risk of PSA failure conferred by increasing preoperative PSA level, a prospective assessment in which men are randomly assigned to BNS versus non-NS if their PSA level exceeds a predetermined cut-point and if all other criteria for NS RP used in this study are met could be considered to determine the impact of preoperative PSA level on future risk of PSA failure. Such a study could ascertain whether the increased risk of PSA failure observed with increasing pre-RP PSA level is due to local persistence of disease that NS may have contributed to or micrometastatic disease, which NS would have no impact on.

Several points require further discussion. First, the decision to perform BNS in our study depended only on the PPB value being low and not on the value of the PSA, as shown in Table 1. Therefore we included an interaction term between PPB and PSA in our multivariable model, given that it is known that increasing PPB and PSA are both associated with increased tumor volume, risk of positive margins, and subsequent PSA failure following NS RP [29–31]. We found that in the multivariable model, the interaction term between PPB and PSA approached significance (*P* = 0.054) and had HR < 1, suggesting that these two factors contribute information regarding the risk of PSA failure interactively. Specifically, this near-significant interaction in our model between PPB and PSA lent support to the hypothesis that an increasing PSA level led to a higher risk of PSA failure, when PPB was less than 2/3. The value of 2/3 was determined by using a mathematical formulation involving taking the logarithms of the AHRs corresponding to PPB, PSA, and PPB*PSA from the Cox interaction model. Second, we know that not all PSA failures lead to clinically significant outcomes such as distant metastasis (DM) or death from prostate cancer. Specifically, as the PSA DT decreases, the risk of observing these clinically significant endpoints increases [32, 33]. Therefore, further follow-up of the data in this study is needed to ascertain if the association between increasing pre-RP PSA level and an increased risk of PSA failure translates to increased risks of DM and death from prostate cancer. Third, studies have not found inferior PSA outcomes when minimally invasive techniques such as robotic or laparoscopic RP as compared to open RP are performed. [34–36]. Therefore, these data using open RP should apply to patients undergoing robotic or laparoscopic RP. Fourth, the median PSA in this study was 4.8 ng/mL, with only 7% of men having a PSA ≥ 10 ng/mL. Therefore, prospective studies in which men who meet all requirements for NS as described in this study but have a PSA in excess of 10 ng/mL should be performed in order to rigorously assess the impact of PSA level as a NS criterion or not on future risk of PSA failure.

Despite these considerations, using criteria for NS that did not include PSA, the use of NS did not increase the risk of PSA failure in a cohort screened with PSA. Therefore it appears that while increasing PSA level was associated with an increased risk of PSA failure, the use of UNS or BNS as compared to non-NS RP was not suggesting that factors other than NS were responsible for the increased risk of PSA failure in men with high pre-RP PSA levels. Whether the increased risk of PSA failure observed with increasing pre-RP PSA level can be reduced by including a predetermined PSA cut-point to select for non-NS requires additional study.

## Figures and Tables

**Table 1:** Comparison of the distribution of clinical and pathologic factors in men selected for bilateral or unilateral versus non-nerve sparing radical prostatectomy.

Clinical factor	Non-nerve sparing (non-NS) *N* = 19	Unilateral nerve sparing (UNS) *N* = 151	Bilateral nerve sparing (BNS) *N* = 187	*P* value comparing non-NS, UNS, and BNS
Median PSA (IQR) (ng/mL)	4.6 [4.0, 10.0]	5.0 [4.0, 6.2]	4.6 [3.5, 6.0]	0.137
Median PPB (IQR) (%)	50.0 [18.2, 70.0]	33.3 [25.0, 50.0]	16.7 [10.0, 25.0]	<0.0001
PSA and PPB category*				
Both > median	8 (10.39%)	50 (64.94%)	19 (24.68%)	<0.0001**
PSA > median PPB ≤ Median	1 (1.04%)	30 (31.25%)	65 (67.71%)	<0.0001**
PSA ≤ median PPB > median	5 (6.25%)	49 (61.25%)	26 (32.50%)	<0.0001**
Both ≤ median	5 (4.81%)	22 (21.15%)	77 (74.04%)	<0.0001**
Median age (IQR) (yrs)	60.2 [52.0, 67.2]	61.4 [57.4, 64.9]	57.8 [52.9, 61.9]	<0.0001
AJCC clinical tumor category				<0.0001**
1c	16 (84%)	109 (72%)	170 (91%)	
2-3	3 (16%)	42 (28%)	17 (9%)	
Biopsy Gleason score				<0.0001**
7 or less	11 (58%)	131 (87%)	187 (100%)	
8 to 10	8 (42%)	20 (13%)	0 (0%)	
AJCC prostatectomy tumor category				<0.0001**
2	12 (63%)	119 (79%)	174 (93%)	
3-4	7 (37%)	32 (21%)	13 (7%)	
Prostatectomy Gleason score				<0.0001**
7 or less	13 (68%)	129 (85%)	187 (100%)	
8–10	6 (32%)	22 (15%)	0 (0%)	
Margin status				<0.0001**
Positive	8 (42%)	18 (12%)	10 (5%)	
Negative	11 (58%)	133 (88%)	177 (95%)	

PSA: prostate-specific antigen; PPB: percentage of positive prostate biopsies; IQR: interquartile range; AJCC: American Joint Commission on Cancer. *Overall, median PPB = 25%; overall, median PSA = 4.8 ng/mL; overall, 7% had PSA ≥ 10 ng/mL. **Fisher exact test *P* value.

**Table 2:** Univariable and Multivariable hazard ratios for clinical factors from the Cox regression analysis for the risk of PSA-failure.

Clinical factor	Number of men	Number of events	Univariable analysis		Multivariable analysis	
			HR (95% CI)	*P* value	AHR (95% CI)	*P* value
Age at diagnosis (years)	357	34	0.99 (0.95, 1.04)	0.814	0.98 (0.92, 1.05)	0.542
PSA (ng/mL)	357	34	1.09 (1.04, 1.15)	0.001	1.09 (1.03, 1.16)	0.005
PPB (%)	357	34	1.04 (1.02, 1.06)	<0.001	1.03 (1.01, 1.05)	0.009
PSA & PPB interaction	357	34	0.999 (0.998, 1.000)	0.177	0.999 (0.998, 1.000)	0.054
Highest Gleason score						
6	208	2	1 (Ref)	—	1 (Ref)	—
7	121	17	15.24 (3.52, 65.99)	<0.001	9.70 (2.17, 43.37)	0.003
8–10	28	15	67.60 (15.45, 295.75)	<0.001	25.01 (5.08, 123.14)	<0.001
AJCC clinical tumor category						
T1c	295	19	1 (Ref)	—	1 (Ref)	—
T2-3	62	15	3.77 (1.91, 7.42)	<0.001	1.49 (0.72, 3.08)	0.283
Type of nerve sparing RP						
BNS	187	3	0.043 (0.011, 0.173)	<0.001	0.407 (0.064, 2.589)	0.341
UNS	151	25	0.473 (0.193, 1.156)	0.101	1.242 (0.355, 4.342)	0.734
Non-NS	19	6	1 (Ref)	—	1 (Ref)	—

HR: hazard ratio; AHR: adjusted hazard ratio; PSA: prostate-specific antigen; PPB: percentage of positive prostate biopsies; AJCC: American Joint Commission on Cancer; RP: radical prostatectomy; BNS: bilateral nerve sparing; UNS: unilateral nerve sparing; Non-NS: non-nerve sparing.
